# Elastic–Plastic Numerical Analysis of the Spinning Process of SA-372 Steel Used in High-Pressure Hydrogen Storage Cylinders (≥100 MPA)

**DOI:** 10.3390/ma16010275

**Published:** 2022-12-28

**Authors:** Ruifeng Yin, Ruidong Fu, Wenlong Wei, Jianfu Gao, Yongjiu Liu, Shuaitao Ge

**Affiliations:** 1 State Key Laboratory of Metastable Materials Science and Technology, Yanshan University, Qinhuangdao 066004, China; 2 College of Materials Science and Engineering, Yanshan University, Qinhuangdao 066004, China; 3 Shijiazhuang Enric Gas Equipment Co., Ltd., No. 169 Yuxiang Street, Shijiazhuang 051430, China; 4 China Classification Society, 40 Dong Huang Cheng Gen Nan Jie, Beijing 100006, China

**Keywords:** SA-372 steel, hydrogen storage cylinder, spinning process, elastic–plastic numerical analysis, hydrogen embrittlement test

## Abstract

Elastic–plastic numerical analysis of the spinning process of SA-372 steel is used in high-pressure hydrogen storage to analyze high-pressure hydrogen storage cylinders with high precision and excellent hydrogen embrittlement resistance. The spinning process of SA-372 steel used to form such a cylinder with a pressure of 100 MPa is investigated through elastic–plastic finite element analysis. The variations in the stress, strain, pressure, temperature, and wall thickness during the spinning processes are comprehensively examined, and the optimized processing parameters are determined based on the numerical analysis results. Finally, these optimal parameters are used to conduct actual spin-forming experiments. The numerical results are found to be in excellent agreement with the experimental results, which verifies the feasibility and effectiveness of the proposed elastic–plastic numerical analysis model for the optimization of spinning process parameters. Furthermore, the hydrogen embrittlement test based on ISO 11114-4:2005 method A proves that the cylinder shoulder has a good hydrogen embrittlement resistance.

## 1. Introduction

Hydrogen energy has been recognized as an effective alternative to fossil fuels for tackling issues such as the global energy crisis, climate change, and environmental pollution [1]. However, since hydrogen is considered to be a hazardous chemical, its production, transportation, refueling, and storage are strictly regulated, which restricts the development of hydrogen and fuel cell technologies in several countries such as China. To boost the practical application of hydrogen as an alternative energy source, it is necessary to build substructures for hydrogen storage and transport [2,3,4,5]. Hydrogenation stations are the core infrastructure for supplying hydrogen to fuel cell vehicles and other energy utilization devices. To achieve fast charging, the ideal hydrogen storage pressure of hydrogenation stations should be above 100 MPa [6,7,8,9,10].

The spinning process, as a non-chip continuous local rotary forming process, is a thermomechanical process [11,12,13], which is widely used to manufacture rotary parts. Compared with the traditional cutting process, the spinning workpiece has several advantages, such as a lightweight structure, high accuracy, and good dynamic balance [14,15,16]. At present, most high-pressure seamless hydrogen storage cylinders are manufactured by spin processing of seamless steel tubes.

The elastic–plastic numerical analysis is an effective method to optimize the processing parameters and has been employed in spinning process simulation [6,17,18,19,20,21]. For example, Xi et al. [22] used the grey correlation method to determine the optimum spinning process parameters based on the analysis of the thinning rate, feed ratio, spinning wheel working angle, and spinning wheel fillet radius. Zhang et al. [17] examined the effect of feed rate on the spinning performance and found that the increase in the feed rate had a minor influence on the contour when the feed rate was not more than 1 mm/r. When the feed rate was greater than 1 mm/r, the contour precision was lost, so the feed rate should be properly raised to control the wall thickness without affecting the contour precision. Li et al. [23,24,25,26,27,28] established a simulation model for the spinning of a Ta-2.5W spherical disc part and analyzed the stress and strain during the spinning process. By examining the difference in the wall thickness after spinning, the influence of feed rate, spinning wheel fillet radius, and spinning wheel working angle on the wall thickness was obtained.

In this study, the spinning process of SA-372 steel utilized to form a high-pressure hydrogen storage cylinder is examined based on the “unit load method” along with complete structural analysis and fatigue lifetime evaluation using the commercial finite element software ABAQUS revision 2021. The unit load method circumvents the limitations of the traditional methods and facilitates a more detailed design using many factors. Furthermore, hydrogen embrittlement experiments were conducted according to the ISO 11114-4:2005 A method. The experimental results reveal that reasonable spinning parameters can effectively improve the hydrogen embrittlement resistance of the shoulder of a high-pressure hydrogen gas cylinder.

## 2. Establishment of the Simulation Model

### 2.1. Simulation Conditions

The spinning process of a high-pressure hydrogen storage cylinder is a complicated dynamic contact process and the plastic zone on the workpiece changes constantly. Therefore, the ABAQUS/explicit dynamic explicit module was used to simulate the process. The SA372 Grade J steel material used for manufacturing high-pressure hydrogen storage cylinders was simulated. The parameters selected for simulating the spinning process of a high-pressure hydrogen storage cylinder are listed in Table 1.

**Figure 1:**
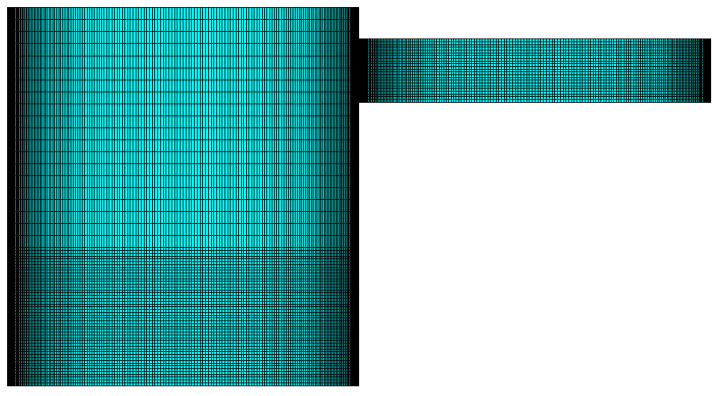
The finite element model of spinning closure.

The blank is only defined as a deformed body in the model. As shown in Figure 1, 8-node hexahedral elements were used for grid division, where the number of tube blank elements was 770,868 and the number of nodes was 833,416. The chuck and rotary wheel are regarded as rigid bodies. Considering that the anisotropy of the material has a minor effect on forming process, the following assumptions are made: (1) The influence of material anisotropy is not considered. (2) The impacts of gravity and inertia are ignored. (3) The simplified treatment of thermal boundary conditions results in a constant temperature in initial conditions.

Based on the display algorithm, the simulation of spin closing can avoid the convergence problem of large deformation and save memory. The Lagrange method was used to describe the finite column formula corresponding to large deformation, and ABAQUS finite element algorithm was used to obtain the results. Through ABAQUS finite element analysis, the influence of spinning temperature and feed rate process parameters on the spin forming of high-pressure hydrogen storage cylinder can be obtained, including equivalent stress distribution, equivalent deformation, spinning pressure, wall thickness, etc.

### 2.2. Influence of Spinning Temperature

The influence of spinning temperature on the spinning process of a high-pressure hydrogen storage cylinder at different temperatures in the range of 800–1100 °C was analyzed under the condition that the other process parameters remain unchanged.

#### 2.2.1. Effect of Spinning Temperature on the Equivalent Effect Distribution

**Figure 2:**
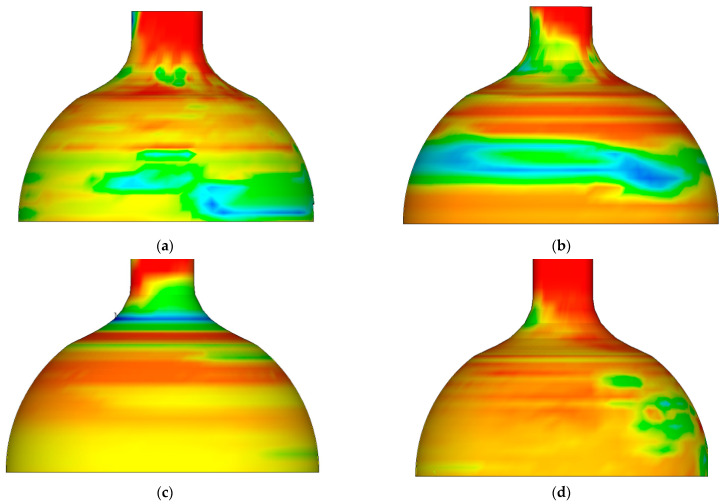
The cloud diagram of equivalent stress distribution after spinning at different temperatures. (**a**) Equivalent stress distribution map at 800 °C. (**b**) Equivalent stress distribution map at 900 °C. (**c**) Equivalent stress distribution map at 1000 °C. (**d**) Equivalent stress distribution map at 1100 °C.

Figure 2 shows the cloud diagram of equivalent stress distribution after spinning at different temperatures. It is clear that the distribution characteristics of equivalent stress are the same at different temperatures from 800–1100 °C. The maximum equivalent stress at each temperature was below the corresponding strength limit of the material, and the billet did not exhibit cracks, fins, or other defects. The peak value of equivalent stress decreased with the increase in temperature.

By sketching and analyzing the equivalent stress distribution diagram after spinning at different temperatures, the distribution characteristics of equivalent stress were uniform in the range of 800~1100 °C. The peak equivalent stress at each temperature point was below the limit of material strength, and no quality defects such as cracks and fins appeared in the billet. The peak value of equivalent stress decreased with increasing temperature.

#### 2.2.2. Effect of Spinning Temperature on the Equivalent Strain

**Figure 3:**
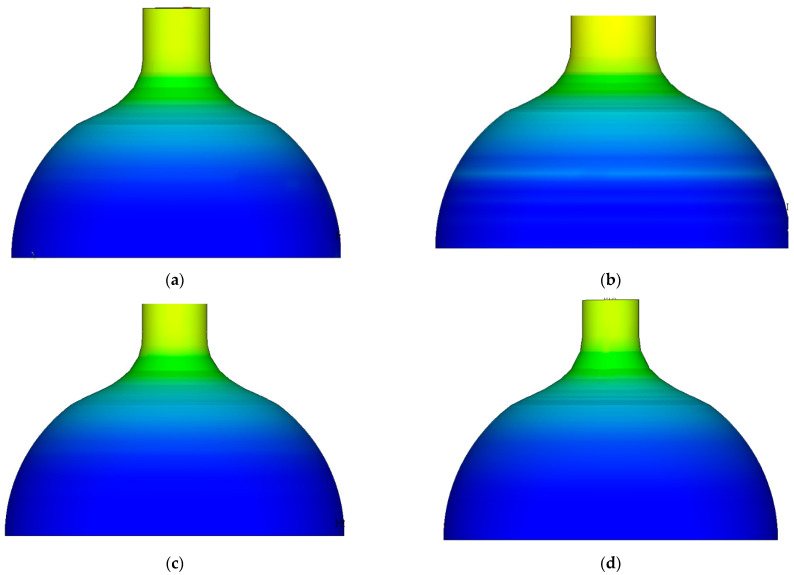
The cloud diagram of equivalent strain distribution after spinning at different temperatures. (**a**) Equivalent strain distribution map at 800 °C. (**b**) Equivalent strain distribution map at 900 °C. (**c**) Equivalent strain distribution map at 1000 °C. (**d**) Equivalent strain distribution map at 1100 °C.

Figure 3 shows the cloud diagram of equivalent strain distribution after spinning at different temperatures. It can be seen that the distribution law of equivalent strain was uniform from 800–1100 °C, and the distribution of equivalent strain was layered along the axis and relatively uniform on the same circumference. At the same temperature point, the peak value of equivalent strain appeared in the straight section of the billet. The higher the temperature, the higher the peak value of equivalent strain.

The distribution law of equivalent strain was the same at 800~1100 °C, and it showed the characteristics of layered distribution along the axis and is uniform in the same circumference. At the same temperature point, the peak value of equivalent strain appeared in the straight section of billet. The higher the temperature, the higher the peak value of equivalent strain.

#### 2.2.3. Effect of Spinning Temperature on Spinning Pressure

**Figure 4:**
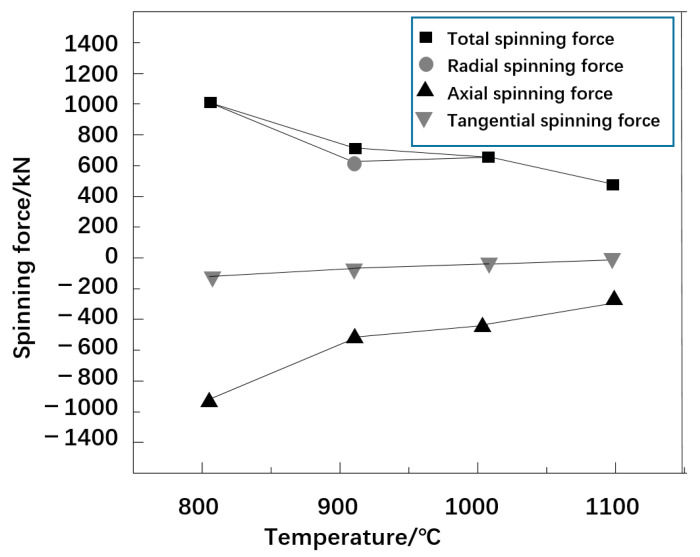
The curve of spinning pressure with temperature.

Figure 4 shows the variation in spinning pressure with spinning temperature. The total, radial, axial, and tangential spinning pressures decrease with the increase in temperature. However, the total spinning pressure and radial spinning pressure changed greatly, while the tangential spinning pressure changed smoothly. The higher the spinning temperature, the lower the spinning pressure. This is because the higher temperature decreases the deformation resistance of the billet metal and strengthens the plasticity, which facilitates the development of the deformation process. This also indicates that the implementation of a hot spinning process for high-pressure hydrogen storage cylinder closure can be effectively promoted by increasing the spinning process temperature, which is in line with the actual production situation.

#### 2.2.4. Influence of Spinning Temperature on the Wall Thickness of a High-Pressure Hydrogen Storage Cylinder

Table 2 shows the maximum and minimum wall thickness values of the high-pressure hydrogen storage bottle after spinning the inlet at different temperatures. It can be seen that with the increase in spinning temperature, the minimum wall thickness decreased, and the maximum wall thickness increased, i.e., the wall thickness at the starting point decreased, and the bottle shoulder became thick. This is mainly because the high-temperature conditions weakened the anti-deformation ability of the metal workpiece and improved its plasticity. The higher the temperature, the easier the metal flow, which results in an increase in the deformation. From the perspective of the influence of spinning temperature on wall thickness, it is helpful to optimize the spinning closing forming effect and the spinning temperature rise process by reducing the spinning temperature under the conditions of a hot deformation process, and the minimum and maximum back wall thicknesses should decrease and increase, respectively.

The analyzed influence of spinning temperature on the stress field, strain field, spinning pressure, and wall thickness of a high-pressure hydrogen storage cylinder during the spinning process suggests that heating temperature must be effectively controlled to reduce the spinning pressure, ensure spinning quality, and conduct spin forming of high-pressure hydrogen storage cylinders under large deformation. On the one hand, if the heating temperature cannot reach the spinning temperature, the elongation of the metal can be poor, which can lead to an increase in the spinning pressure. Based on the comprehensive simulation results and the performance change of SA372 Grade J steel at high temperatures, the appropriate temperature range of 850–950 °C was finally selected for the spinning process.

### 2.3. Effect of Feed Ratio on Spin Forming

The spin-forming process of high pressure hydrogen storage cylinder with a feed ratio of 1.5 mm/r, 2.0 mm/r, and 2.5 mm/r was simulated and analyzed under a spinning temperature of 900 °C and the same process parameters.

#### 2.3.1. Effect of Feed Ratio on Stress Field Distribution

**Figure 5:**
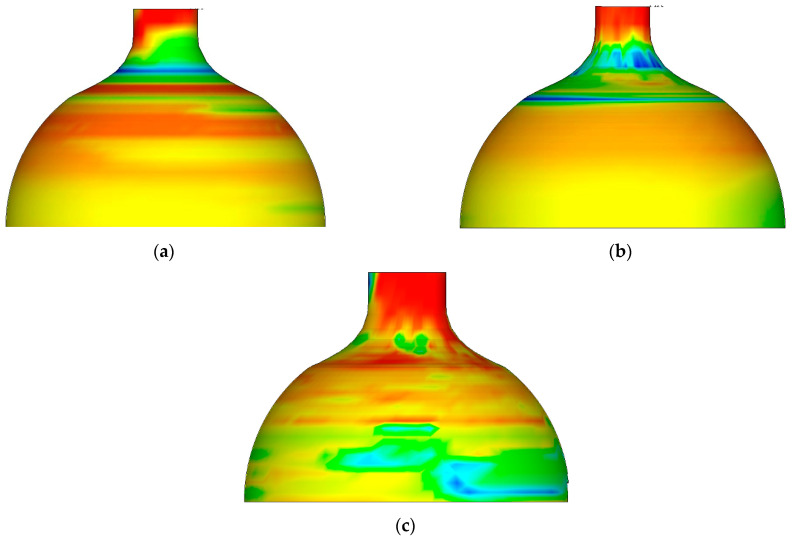
The Cloud diagram of equivalent stress distribution with different feed ratios. (**a**) Feed ratio is 1.5 mm/r. (**b**) Feed ratio is 2.0 mm/r. (**c**) Feed ratio is 2.0 mm/r.

The cloud diagram of equivalent stress distribution after spinning forming with different feed ratios is shown in Figure 5. It can be seen that the distribution law of equivalent stress was the same after the deformation of the billet, and the increase in feed ratio did not have an obvious effect on the change in stress size. With the increase in feed ratio, the peak value of equivalent stress was also slightly increased.

#### 2.3.2. Influence of Feed Ratio on Strain Field Distribution

**Figure 6:**
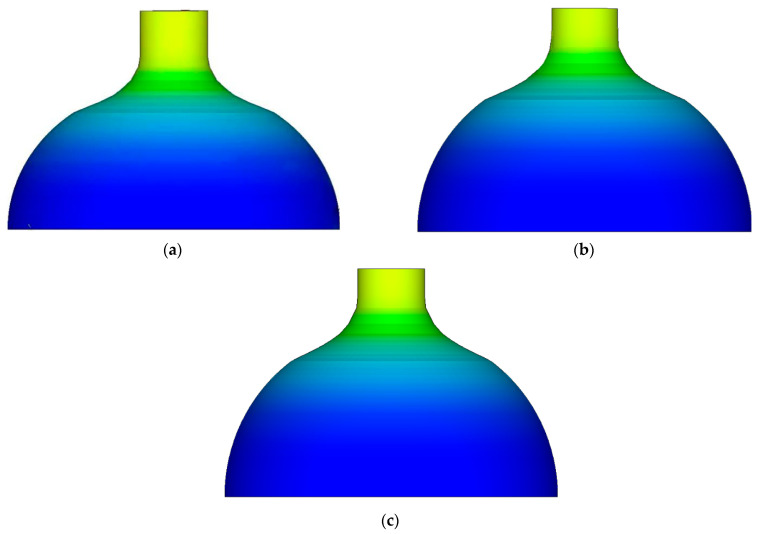
The Cloud diagram of equivalent strain distribution with different feed ratios. (**a**) Feed ratio is 1.5 mm/r. (**b**) Feed ratio is 2.0 mm/r. (**c**) Feed ratio is 2.0 mm/r.

The equivalent strain distribution cloud diagram after the formation of spinning with different feed ratios is shown in Figure 6. It can be seen that the equivalent strain distribution law of the billet was basically the same after the feed ratio was 1.5 mm/r, 2.0 mm/r, and 2.5 mm/r. The equivalent strain was distributed in layers along the axial direction, and the distribution was relatively uniform on the same circumference. The straight section of the tube blank is the place where the peak value of equivalent stress appears under each feed ratio. With the increase in the feed ratio, the equivalent strain did not change significantly but showed a tendency of slightly increasing.

#### 2.3.3. Effect of Feed Ratio on Rotation

**Figure 7:**
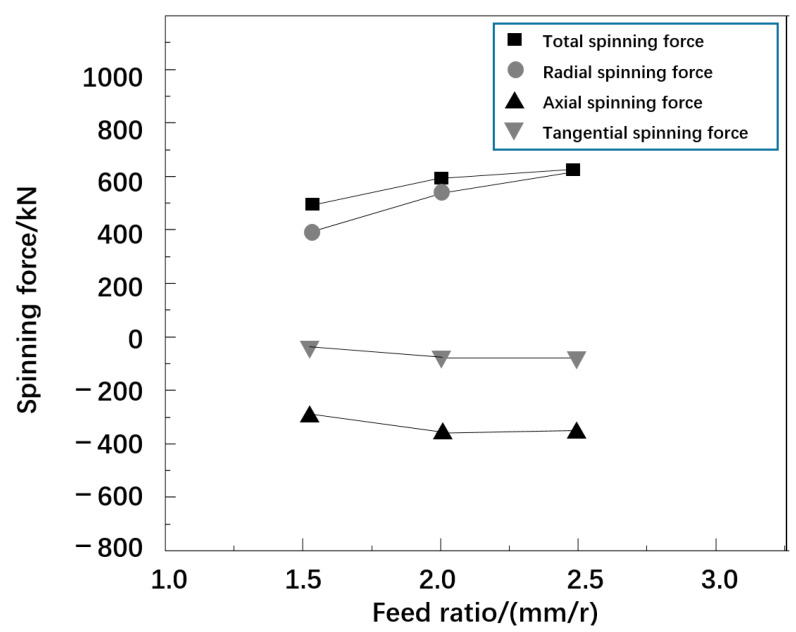
The variation curve of spinning force with the feed ratio.

The curve of maximum spinning pressure with the increase in feed ratio is shown in Figure 7. The total rotational pressure increased from 588.2 kN to 754.3 kN, i.e., an increase of 166.1 kN; the radial rotational pressure increased from 531.0 kN to 785.3 kN, i.e., an increase of 197.1 kN; the axial spinning force increased from −423.1 kN to −478.6 kN, i.e., an increase of 55.5 kN; the tangential rotation pressure increased from −67.3 kN to −83.4 kN, i.e., an increase of 16.1 kN. It can be seen that with the increase in feed ratio, the total spinning pressure and three-way spinning pressure both show an increasing trend. The causes of the above phenomena were analyzed. The main reason is that in the stage of spinning operation, the track touched by both the rotating wheel and the billet was a single spiral line. In the stage of increasing feed ratio, the area where the spirals coincided with each other tended to decrease, and more metal billets changed in shape, which induced and promoted the increase in spinning pressure.

#### 2.3.4. Effect of Feed Ratio on Cylinder Wall Thickness

The maximum and minimum wall thicknesses of cylinders with a feed ratio of 1.5 mm/r, 2.0 mm/r, and 2.5 mm/r after spinning are described in Table 3. With the increase in feed ratio, the minimum wall thickness at the starting point of the cylinder became thinner, while the wall thickness at the maximum wall thickness of the cylinder shoulder increased, and the increasing trend of wall thickness was more obvious with the increase in feed ratio. Because the feed ratio increased, the deformation speed of the billet metal increased, and the shrinkage metal caused the billet wall thickness to accumulate from thin to thick. In this case, the forward flow of the same section of metal became slower, and the billet wall thickness increased faster.

In General, the feed ratio had little effect on the increase or decrease in the billet wall’s thickness. The principle of selecting the ratio of rotating wheel feed is to maximize production efficiency while satisfying the deformation condition and quality requirement.

## 3. Spinning Experiment

**Figure 8:**
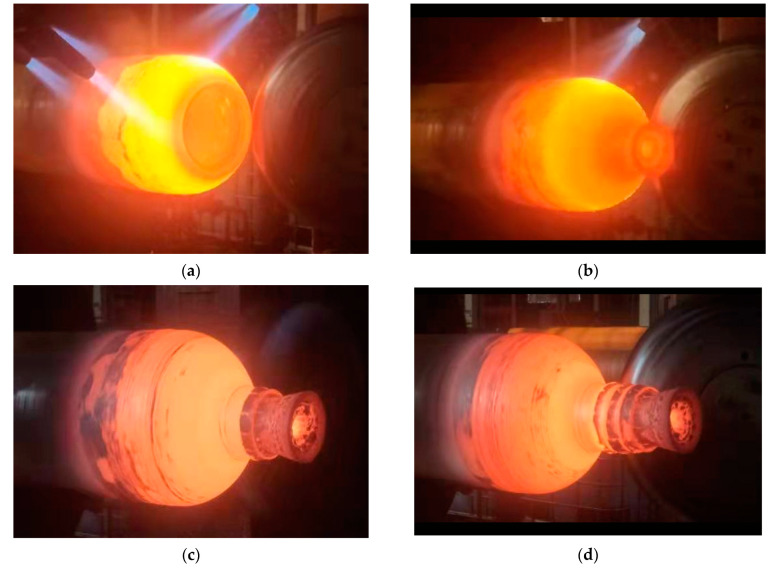
Spinning forming experiment process. (**a**) Spinning process 1. (**b**) Spinning process 2. (**c**) Spinning process 3. (**d**) Spinning process 4.

Based on the finite element simulation analysis, the optimized spinning parameters were obtained: the spinning temperature was 900 °C, the roller feed rate was 2 mm/s. Then, these parameters were used to verify the optimization results obtained using ABAQUS simulation, the high-pressure hydrogen storage cylinder was formed by using nine passes in the spinning process, and the cylinder spinning experiment process with good forming quality was conducted using the optimized process parameters as shown in Figure 8.

## 4. Hydrogen Embrittlement Test after Spinning

### 4.1. Test Principle

A mounted disc-shaped test piece was subjected to an increasing gas pressure at a constant rate to facilitate bursting or cracking. The hydrogen embrittlement effect was demonstrated by comparing the hydrogen rupture pressure PH2 with the helium rupture pressures PHe, where helium was chosen as a reference gas. Specifically, the ratio PHe/PH2 was determined. The lower this ratio, the better the behavior of steel in the presence of hydrogen. This ratio was dependent on the pressure rise rate, which remained constant during the entire test.

The test results were interpreted considering that the material embrittlement index was equal to the maximum value of the ratio PHe/PH2 and that the material was suitable for compressed hydrogen cylinders if the index was less than or equal to 2.

### 4.2. Test Conditions and Procedure

The tests were conducted with high-purity hydrogen (>99.9995%) and helium (H20 < 3 μL/L) for different pressure rise rates ranging between 0.1 bar/min to 1000 bar/min. The minimum value of the rupture pressure was determined when the later pressure rates were employed. This minimum value was used to run the remaining tests. Generally, six helium tests and nine hydrogen tests (i.e., 15 tests in total) are considered to be sufficient for a thorough material evaluation.

**Figure 9:**
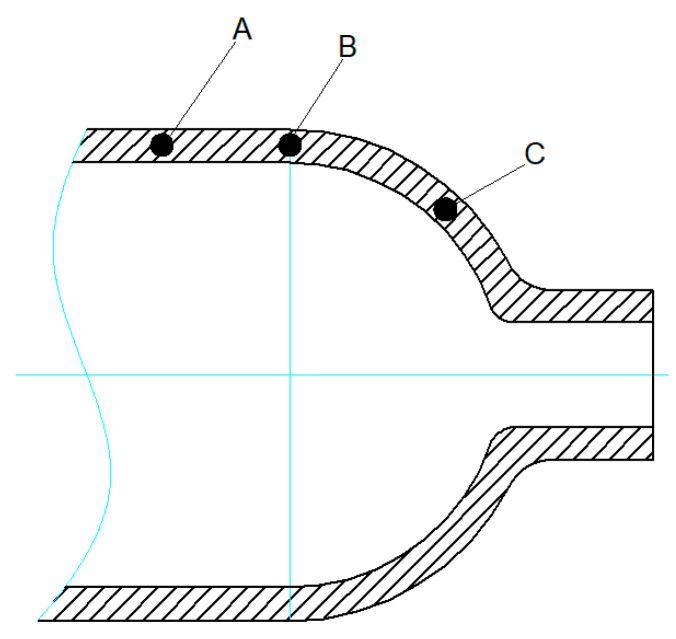
Sampling locations.

**Figure 10:**
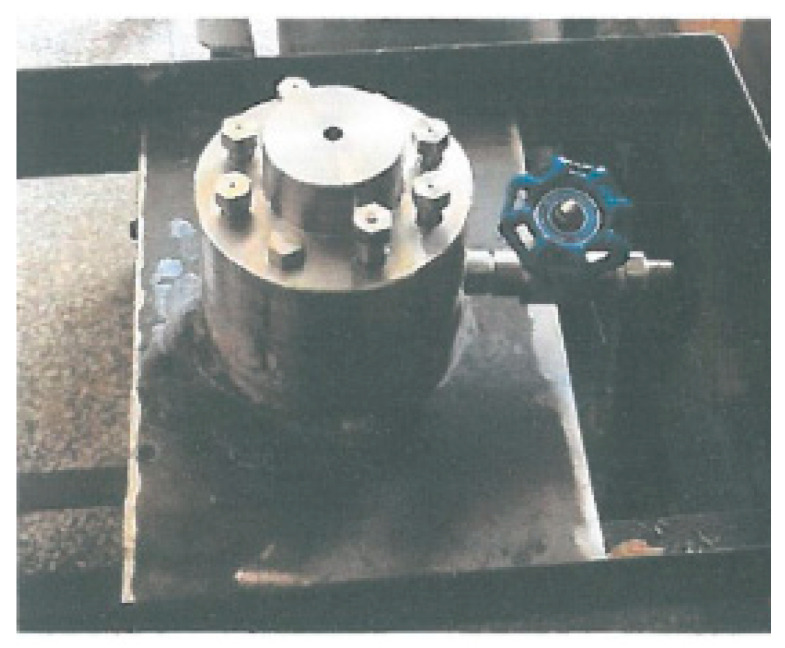
Test equipment.

The sampling locations after spinning are shown in Figure 9. The sampling direction is circular, and the sampling locations are the shoulder and joint of the cylinder body and the junction between the body and shoulder. The sampling depth is 1/2 wall thickness from the surface. All the tests were conducted at room temperature (20 °C). A snapshot of the test equipment is shown in Figure 10.

Disc sampling (A, B, and C) is shown in Figure 9 after spinning the cylinder. The sampling direction is circular. The sampling positions are the cylinder body (A), the joint of the cylinder body (B) and shoulder (C). The sampling depth is 1/2 wall thickness from the surface.

### 4.3. Test Results

The corrected rupture pressures are plotted against the mean pressure rise rate (actual rupture pressure divided by the test duration; expressed in bar/min), and for each hydrogen test, the ratio Pr′_He_/Pr′_H2_ was calculated. Here, Pr′_He_ is the theoretical helium rupture pressure corresponding to the same pressure rise rate as that for the hydrogen test, which is calculated from the regression equation of the corrected helium rupture pressure. Pr′_H2_ is the corrected hydrogen rupture pressure. The variation in the ratio Pr′_He_/Pr′_H2_ as a function of the pressure rise rate is shown in Table 4

The test results were interpreted considering that the material embrittlement index is the maximum value of the ratio Pr′_He_/Pr′_H2_ and that the material is suitable for compressed hydrogen cylinders if the index is less than or equal to 2.

The hydrogen embrittlement test results after spinning show that the material has excellent hydrogen embrittlement property and can meet the requirements of international gas cylinder standard ISO 11114-4:2005.

## 5. Conclusions

An elastic–plastic numerical model was established for the steel spinning process to form a high-pressure hydrogen storage cylinder based on the stress–strain curves and the finite element software ABAQUS. The main results of the study are summarized as follows:

1. With the increase in temperature, the maximum value of the equivalent stress gradually decreases, the maximum value of the equivalent strain gradually increases, and the total rotational pressure, radial, axial and tangential rotational pressure decreases.
2. With the increase in feed ratio, the total spin pressure and the three-way spin pressure both show an increasing trend, and the equivalent strain slightly increases.
3. The optimized processing parameters were determined based on the numerical analysis results. The pinning temperature and the roller feed ratio were 1000 °C and 2.0 mm/s, respectively.
4. The hydrogen embrittlement test after spinning was performed based on the ISO 11114-4:2005 method using the optimized process parameters. The results revealed that the optimized spinning parameters could effectively improve the hydrogen embrittlement resistance of the shoulder of the high-pressure hydrogen storage cylinder.

## Figures and Tables

**Table 1:** The simulated parameters.

Outer diameter of cylinder	406 mm
Thickness of cylinder	38 mm
Density	7750 kg/m^3^
Poisson’s ratio	0.3
Elastic modulus	202 GPa

**Table 2:** The maximum and minimum wall thickness of cylinders after spinning at different temperatures.

Temperature 1100 °C	Minimum Wall Thickness (mm)	Maximum Wall Thickness (mm)
800	37.4	44.5
900	37.6	45.3
1000	38.0	46.2
1100	38.4	47.5

**Table 3:** The maximum and minimum wall thickness of cylinders after spinning at Feed ratio.

Feed Ratio (mm/r)	The Minimum Wall Thickness (mm)	The Maximum Wall Thickness (mm)
1.5	39.2	44.1
2.0	38.6	46.5
2.5	37.8	47.7

**Table 4:** Ratio Pr′_He_/Pr′_H2_ for different samples.

Sample No.	A3	A4	A5	B3	B4	B5	C3	C4	C5
Pressure rise rate (bar/min)	62.2	30.1	3.7	355.4	239.2	20.0	15.1	6.0	157.4
Corrected helium rupture pressures Pr′_He_ (bar)	623.9	617.0	596.7	640.7	636.9	613.0	610.4	601.5	632.9
Corrected hydrogen rupture pressure Pr′_H2_ (bar)	380.3	379.0	450.2	546.9	428.6	328.0	347.3	332.7	478.3
Pr′_He_/Pr′_H2_	1.6	1.6	1.3	1.2	1.5	1.9	1.8	1.8	1.3
Acceptance level	Hydrogen embrittlement index ≤ 2								

## Data Availability

Not applicable.
